# Investigation and intervention of psychological factors among migrant workers in electronics enterprises in Kunshan, China

**DOI:** 10.1186/s12889-026-27359-4

**Published:** 2026-04-14

**Authors:** Xianping Song, Xukun Wan, Guohua Qian, Quan Chen, Hao Chen, Baoli Zhu, Zhimin Tong, Huanxi Shen

**Affiliations:** 1 Kunshan Center for Disease Prevention and Control, Suzhou, China; 2 Kunshan Integrated Traditional Chinese and Western Medicine Hospital, Suzhou, China; 3 Jiangsu Provincial Center for Disease Control and Prevention, Institute of Occupational Disease Prevention, Nanjing, China

**Keywords:** Migrant electronics workers, Mental health, SCL-90-R, Psychological intervention

## Abstract

**Background:**

Following the COVID-19 pandemic, there has been a notable decrease in market share and production capacity, which has coincided with an increase in mental health challenges, particularly among migrant electronics workers in China.

**Methods:**

To assess their psychological well-being, the Symptom Check list 90 Revised (SCL-90-R) was administered to 1,544 migrant electronics workers in Kunshan. Out of the respondents, 280 individuals who exhibited SCL-90-R scores exceeding the national average participated in an intervention program. This program comprised psychological counseling, peer support, and knowledge training sessions focused on effective communication with peers.

**Results:**

The findings indicated that migrant workers reported higher levels of obsessive-compulsive symptoms, interpersonal sensitivity, depression, and anxiety compared to the national average. The intervention program was associated with a significant reduction in SCL-90-R scores, decreasing from 153.33 ± 20.91 to 121.9 ± 11.43(*P* < 0.05).

**Conclusions:**

The study concludes by emphasizing the importance of integrating occupational health considerations with psychological well-being in mental health interventions.

**Supplementary Information:**

The online version contains supplementary material available at 10.1186/s12889-026-27359-4.

## Introduction

 Migration and mobility are important promoting factors for global economic development; however, they also bring about a series of problems, with the mental health issues of immigrants being even more significant. Studies of migrants in Switzerland, France, Italy, Venezuela and other countries have shown increased mental health issues such as depression, anxiety and post-traumatic stress after migration, which can be attributed to geographical discrimination and negative environmental impacts, as well as unequal access to health care [1–3].

China has the largest working population of 776 million in the world, with more than 200 million exposed to occupational hazards [4]. The majority of workers spend over half of their lives in their careers. With the economic transformation and upgrading, occupational disease hazards have become diverse but complex. Traditional occupational disease hazards have not been fundamentally controlled, and work-related diseases, such as mental disorders caused by social and psychological factors, have become increasingly prominent in China [5]. Occupational hazards not only cause physical and physiological harm to the exposed population but also affect their mental and emotional states. Kunshan, an industrially developed city in the Yangtze River Delta, hosts over 1,000 electronic processing enterprises. More than 100,000 migrant workers in this city suffer various occupational hazards [6]. After the COVID-19 pandemic, some enterprises have seen a decline in market share and production capacity due to the macro-factors, resulting in reduced employment and wages. Particularly, employees in electronics processing enterprises, who generally comprise younger migrant workers, are engaged in work characterized by monotony, shift work, and assembly line operations. These factors, combined with job instability, unmet wage expectations, and lack of social networks and social support, can have adverse effects on the mental health of migrant workers [7, 8].

Research on the mental health of migrant workers in the electronics industry mainly focuses on general mental health status, the incidence of mental illness, and the influencing factors [9]. Studies have demonstrated that migration is a process full of competition and pressure. Differences in language and culture, changes in social relations, etc., contribute to the poorer mental health of migrant workers than the general population [10–11]. The great changes in the market since the COVID-19 pandemic have significantly impacted employees’ employment and psychology. At present, no relevant studies have been conducted on the mental health problems of migrant workers in the electronics industry in Kunshan in the post-pandemic period.

Mental health can be intervened in many ways. For instance, psychological counseling has proven an effective solution to psychological problems [12]. In addition, peer support, which includes mutual assistance and professional training, also significantly improves the mental health and well-being of vulnerable groups. Studies have shown that peer support substantially boosts mental health by offering emotional comfort [13].

To measure mental health, this study used the Symptom Check List 90 Revised (SCL-90-R), developed by Derogatis et al. [14]. The SCL-90-R, as a famous mental health assessment scale, is the most widely used outpatient examination scale for mental disorders and mental illnesses. This study used on-site questionnaires (SCL-90-R) to investigate the mental state, psychological needs and coping methods of urban migrant workers. This study also integrated existing studies to explore the mental health intervention policies for urban migrant workers in electronic enterprises. The goal was to understand their mental health level and improve their coping ability with psychological problems.

## Methods

### Subjects

Cluster sampling was used to select ten large and medium-sized electronics enterprises in Kunshan. The primary occupational hazards identified in these enterprises included noise, dust, Tin (IV) oxide, indium, DMF (N, N-Dimethylformamide), etc. The assessment of occupational hazard exposure was based on enterprise occupational hygiene records and self-reported exposure questionnaires administered to workers. Within each enterprise, a simple random sample of 100–200 front-line migrant workers was drawn from the complete employee roster provided by the human resources department. A total of 1,800 workers were initially invited to participate. Of these, 1,600 provided informed consent and completed the survey, yielding a response rate of 88.9%. The primary reason for non-participation was lack of available time during work shifts. Migrant workers refer to “laborers who enter the city from outside the county (city) to engage in non-agricultural labor for more than six months, with the city as their usual residence, and non-agricultural income as their main income.” The mental health status of the participants was investigated using a situation survey form and the SCL-90-R. All respondents participated voluntarily with informed consent.

### Questions

The situation survey form comprised 29 items on basic situation, employment situation, income distribution, and social activities (An additional file shows this in more detail [see Additional file 1]). The SCL-90-R, which is currently the most widely used outpatient examination scale for mental disorders and mental illnesses [15, 16], consists of 90 items and 9 sub-scales: somatization, obsession, sensitivity, depression, anxiety, hostility, phobia, paranoia, and psychosis. It employs a 5-level scoring system, where a higher score indicates more severe psychological symptoms: 1 = none, 2 = mild, 3 = moderate, 4 = severe, 5 = very severe. The SCL-90-R contains of 9 latent factors, with a factor average score of 2 or higher indicating varying degrees of psychological problems (More detail showed in Additional file 2). The SCL-90-R scale has undergone rigorous reliability and validity tests to ensure the objectivity and accuracy of the evaluation results.

### Intervention

#### Intervention measures

The first psychological evaluation of the subjects before the intervention was used as pre-test data. The intervention included the following components:

1. WeChat mutual aid group: Subjects were randomly assigned to groups of 20 people, each including at least one investigator and one psychological counsellor. Offline activities for the WeChat mutual-aid groups were organized once per month. The main purpose of the group was to encourage open communication and organize regular offline activities.
2. Educational courses: a total of 20 weeks sessions were conducted, each class lasting 1 hour. The course included medical psychology and occupational health education. Theoretical courses were primarily conducted online, whereas practical courses took place offline in small groups. Theoretical courses employed case-based and heuristic teaching; during the experimental class, participants watched psychological films or participated in fun competitions, followed by group discussions that integrated the theoretical knowledge learned; participants were also guided to use psychological software for personality tests to understand their personality traits.
3. Establishment of good employment relationships: Efforts were made to coordinate employee relationships. Employers regularly and irregularly were encouraged to communicate and exchange with employees, listen to their opinions, answer their questions, and help alleviate any negative emotions.

One week after all the courses, a second evaluation was performed on the subjects as post-test data. Intervention fidelity was ensured by monitoring attendance (average rate: 92%), participation in monthly offline activities (88%), and adherence to a standardized protocol. Anonymous feedback surveys indicated high participant satisfaction (mean rating: 4.2/5).

#### Intervention subjects

A total of 307 people with a score of more than 130 (i.e., above the Chinese norm [17]) on the SCL-90-R survey were selected. This cut-off represents a normative threshold (approximately 1 SD above the national mean) commonly used in epidemiological research to identify individuals with elevated psychological symptoms. After excluding invalid questionnaires due to manual errors and those who did not volunteer or were unable to accept the entire intervention treatment, 280 participants were selected. The sample size was determined pragmatically, including all eligible workers who consented to participate, consistent with the exploratory nature of the study and the ethical commitment to offer intervention to all at-risk individuals. A 6-month intervention treatment was carried out for this group.

### Statistical analysis

The situation survey form and SCL-90-R data were entered into the EpiData database using the double-entry method. Descriptive and logistic regression analyses were conducted using the R Programming Language 4.0.0. Student’s t-test and one-way ANOVA were performed to compare continuous variables between two and three groups, respectively. Binary logistic regression analysis was used to analyze influencing factors. Categorical predictors in the regression model (e.g., age group, hazard exposure level) were coded using dummy variables, with reference categories specified. The model’s goodness-of-fit was assessed using the Hosmer-Lemeshow test, and multicollinearity among predictors was examined by calculating Variance Inflation Factors (VIFs), all of which were below 2.0, indicating no critical collinearity.

### Quality control

Unified training was provided for survey and data entry personnel. A pre-survey was conducted to ensure the quality of the survey. Before the evaluation, the staff clearly explained the evaluation method and requirements to the participants, who were then asked to complete the evaluation independently. After the survey was completed, the supervisor reviewed corrected and summarized the survey forms. Missing values were handled either by deletion or by imputing the average value (when five or fewer questions were left unanswered). Invalid questionnaires were defined as those with > 10% missing items, inconsistent responses, or clear patterns of bias.

## Results

### Comparison of SCL-90-R factors with Chinese norms

A total of 1544 valid questionnaires were collected from 1600 migrant workers in electronics enterprises in Kunshan. The data followed an approximately normal distribution. The respondents included 1,061 males and 483 females, aged 17–52 (mean age: 27.55 ± 5.82 years), with an average working experience of 3.82 ± 0.58 years.

The total mental health score of migrant workers, based on the SCL-90-R, was 135.18±40.51 on the SCL-90-R, significantly higher than the Chinese norm [17] (general population: 129.96±38.76). A comparison of each factor of SCL-90-R with the Chinese norm indicated the scores for obsessive-compulsive symptoms, interpersonal sensitivity, depression, and anxiety were statistically significantly higher than the norm. Conversely, the scores for somatization, phobia, paranoia, and psychosis were lower than the Chinese norm. Hostility scores were also lower than the Chinese norm, though this difference was not statistically significant. The results are shown in Table 1.

**Table 1:** Comparison of SCL-90-R between migrant workers and the Chinese norm

	SCL-90-*R* (‾x ± s)	Chinese norm(‾x ± s) [17]	t	*P*
Total score	135.18 ± 40.51	129.96 ± 38.76	19.044	< 0.001
Somatization	1.34 ± 0.36	1.37 ± 0.48	-5.185	< 0.001
Obsession	1.73 ± 0.45	1.62 ± 0.58	12.314	< 0.001
Sensitivity	1.90 ± 0.38	1.65 ± 0.61	30.807	< 0.001
Depression	1.68 ± 0.39	1.50 ± 0.59	21.634	< 0.001
Anxiety	1.55 ± 0.37	1.39 ± 0.43	14.770	< 0.001
Hostility	1.47 ± 0.42	1.48 ± 0.56	-1.377	0.081
Phobia	1.19 ± 0.34	1.23 ± 0.41	-4.053	< 0.001
Paranoia	1.30 ± 0.37	1.43 ± 0.57	-18.719	< 0.001
Psychosis	1.25 ± 0.34	1.29 ± 0.42	-7.759	< 0.001

### Comparison of SCL-90-R in occupational groups of different genders

There were statistically significant differences between occupational groups of different genders in terms of the anxiety and phobia factors. Anxiety levels were higher in females than in males, while phobia levels were higher in males than in females. However, no statistically significant differences were found in other factors. The results are shown in Table 2.

**Table 2:** A between-gender comparison of the SCL-90-R total and subscale scores among migrant workers

	Male (‾x ± s)	Female(‾x ± s)	t	*P*
*N*	1061	483	/	/
Total score	135.32 ± 30.81	134.95 ± 29.93	0.218	0.827
Somatization	1.34 ± 0.38	1.33 ± 0.24	0.415	0.678
Obsession	1.73 ± 0.45	1.74 ± 0.61	-0.649	0.516
Sensitivity	1.91 ± 0.39	1.90 ± 0.37	0.795	0.426
Depression	1.67 ± 0.40	1.68 ± 0.38	-0.635	0.526
Anxiety	1.53 ± 0.35	1.57 ± 0.38	-3.088	0.013
Hostility	1.48 ± 0.40	1.47 ± 0.45	0.801	0.423
Phobia	1.22 ± 0.35	1.18 ± 0.34	2.317	*0.021*
Paranoia	1.30 ± 0.38	1.29 ± 0.35	1.508	0.132
Psychosis	1.26 ± 0.35	1.25 ± 0.32	1.408	0.159

### Comparison of SCL-90-R in occupational groups with different lengths of service

The SCL-90-R total score of mental health and various factors of occupational groups with different working years were compared. Workers with more than 5 years of working experience showed significantly higher scores in total mental health, somatization, obsessive-compulsive symptoms, depression, and hostility compared to workers with less than 5 years of experience. However, other factors exhibited no statistical significance, as shown in Table 3.

**Table 3:** A comparison of the SCL-90-R total and subscale scores across seniority groups among migrant workers

	< 5 years(‾x ± s)	≥5years(‾x ± s)	t	*P*
*N*	1048	496	/	/
Total score	130.60 ± 31.19	136.43 ± 33.39	-23.226	*< 0.001*
Somatization	1.30 ± 0.37	1.37 ± 0.40	-3.461	*0.001*
Obsession	1.70 ± 0.46	1.76 ± 0.50	-2.026	*0.043*
Sensitivity	1.89 ± 0.39	1.91 ± 0.40	-1.003	0.316
Depression	1.64 ± 0.40	1.72 ± 0.42	-2.480	*0.013*
Anxiety	1.53 ± 0.37	1.57 ± 0.43	-1.722	0.085
Hostility	1.44 ± 0.43	1.50 ± 0.45	-2.177	*0.030*
Phobia	1.20 ± 0.35	1.19 ± 0.35	0.263	0.792
Paranoia	1.31 ± 0.39	1.28 ± 0.38	0.982	0.326
Psychosis	1.23 ± 0.33	1.27 ± 0.42	-1.518	0.130

### Comparison of SCL-90-R in different types of occupational hazard factors exposure

A total of 399(25.84%) participants were not exposed to risk factors, and 558(36.14%)、398(25.78%)、112(7.25%)、77 (4.99%) respondents were exposed to 1, 2, 3 and more than 3 types risk factors respectively. A comparison of the SCL-90-R among occupational groups with different occupational hazard factors exposure indicated that there were statistically significant differences in the total scores and various factors except the phobia. In general, the more types of risk factors exposed, the higher the score of each SCL-90-R factor. The results are shown in Table 4.

**Table 4:** Comparison of SCL-90-R in different types of occupational hazard factors exposure

	No hazard factors exposure	Exposed to 1 hazard factor	Exposed to 2 hazard factors	Exposed to 3 hazard factors	Exposed to more than 3 risk factors	F	*P*
Total score	131.46 ± 32.09	134.10 ± 37.43	135.71 ± 38.13	140.37 ± 39.50	151.82 ± 42.14	5.935	*0.001*
Somatization	1.24 ± 0.31	1.28 ± 0.32	1.36 ± 0.44	1.38 ± 0.48	1.43 ± 0.45	6.389	*0.001*
Obsession	1.49 ± 0.38	1.63 ± 0.62	1.84 ± 0.65	1.89 ± 0.68	2.05 ± 0.80	2.447	0.045
Sensitivity	1.79 ± 0.33	1.82 ± 0.37	1.86 ± 0.43	1.99 ± 0.46	2.13 ± 0.89	2.936	0.020
Depression	1.50 ± 0.40	1.69 ± 0.64	1.75 ± 0.73	1.76 ± 0.74	1.91 ± 0.78	7.388	*0.001*
Anxiety	1.49 ± 0.39	1.56 ± 0.40	1.60 ± 0.49	1.65 ± 0.51	1.69 ± 0.63	4.962	*0.001*
Hostility	1.42 ± 0.35	1.46 ± 0.34	1.47 ± 0.45	1.61 ± 0.50	1.87 ± 0.59	8.456	*0.001*
Phobia	1.11 ± 0.31	1.21 ± 0.32	1.24 ± 0.39	1.24 ± 0.40	1.21 ± 0.28	0.616	0.651
Paranoia	1.21 ± 0.34	1.31 ± 0.40	1.45 ± 0.39	1.45 ± 0.45	1.46 ± 0.50	5.243	*0.001*
Psychosis	1.22 ± 0.33	1.25 ± 0.38	1.40 ± 0.42	1.42 ± 0.45	1.58 ± 0.48	4.388	0.002

### Comparison of SCL-90-R in occupational groups of different age groups

Subjects were divided into three groups based on age:17–35 years old, 35–45 years old and 45 years old and above. The comparison of the SCL-90-R mental health score and various factors among occupational populations in the three groups showed statistically significant differences in the total score, somatization, depression and paranoia factors. Further pairwise comparison indicated that these four factors scoring were statistically significant in the 17–35 years group compared with the other two groups. In contrast, between the 35–45 years old and the elderly group, only somatization showed statistical significance, while the others did not exhibit statistical significance, as shown in Table 5.

**Table 5:** A comparison of the SCL-90-R total and subscale scores across age groups among migrant workers

	17–35 years(‾x ± s)	35–45 years(‾x ± s)	≥45 years(‾x ± s)	F	*P*
Total score	132.78 ± 28.59	139.00 ± 33.32	136.71 ± 36.63	4.138	*0.019*
Somatization	1.28 ± 0.33	1.34 ± 0.42	1.38 ± 0.43	9.277	*0.001*
Obsession	1.72 ± 0.42	1.75 ± 0.48	1.73 ± 0.56	1.810	0.164
Sensitivity	1.91 ± 0.36	1.87 ± 0.41	1.89 ± 0.44	2.385	0.092
Depression	1.68 ± 0.37	1.71 ± 0.43	1.66 ± 0.44	4.925	*0.007*
Anxiety	1.54 ± 0.35	1.57 ± 0.40	1.53 ± 0.39	1.397	0.248
Hostility	1.46 ± 0.40	1.47 ± 0.44	1.43 ± 0.46	2.775	0.063
Phobia	1.19 ± 0.34	1.19 ± 0.34	1.22 ± 0.40	0.318	0.728
Paranoia	1.28 ± 0.35	1.32 ± 0.40	1.25 ± 0.41	3.677	*0.026*
Psychosis	1.24 ± 0.33	1.24 ± 0.35	1.27 ± 0.41	1.152	0.316

### Binary logistic regression analysis of mental health score on migrant workers in electronic enterprises

The binary logistic regression analysis was conducted with the dependent variable of whether the total mental health score of the participants exceeded the Chinese norm, with the statistically significant factors from Table 6 as independent variables. The method selects forward (LR) in the model, Hosmer-Lemeshow fit test *P* > 0.05, indicating that this model fits well. The results showed that age (≥45 years), working age (≥5 years) and exposure to three or more risk factors were independent influencing factors (*P* < 0.05), as shown in Table 6.

**Table 6:** Risk factors affecting migrant workers as measured by the SCL-90-R total scores

Variables	β	Standard error	Wald χ2	*P*	RR	95% CI
Gender	-0.134	0.160	0.704	0.401	0.874	0.639–1.196
Age(years)			5.011	0.003		
17–35					1	
35–45	0.078	0.112	0.445	0.508	1.079	0.861–1.297
≥45	0.505	0.157	3.173	0.013	1.379	1.169–1.589
Marital status	0.229	0.172	1.766	0.184	1.257	0.897–1.761
Working years	0.339	0.114	9.564	0.001	1.427	1.157–1.697
Exposure to occupational hazard factors			14.346	0.014		
No hazard factors are exposed					1	
Exposure to 1 hazard factor	0.422	0.255	2.731	0.098	1.525	0.925–2.514
Exposure to 2 hazard factors	0.417	0.264	2.489	0.115	1.517	0.904–2.547
Exposure to 3 hazard factors	0.880	0.320	7.573	0.006	2.412	1.288–4.515
Exposure to more than 3 risk factors	1.193	0.390	9.353	0.020	3.296	1.535–7.079

Dependent variable assignment: whether the total SCL-90-R score exceeds the Chinese norm: No = 0, yes = 1; Argument assignment: Gender: Male = 0, female = 1; Age (Dummy variable):17–35 years = 0(reference),36–45 years = 1.45 years = 2; Marital status: married = 0, unmarried = 1; Working years: Working years < 5 = 0, working years ≥5 = 1; Exposure to occupational hazard factors: No hazard factor are exposed = 0(reference), Exposure to 1 hazard factor = 1, Exposure to 2 hazard factors = 2, Exposure to 3 hazard factors = 3, Exposure to more than 3 risk factors = 4. *VIFs* Variance Inflation Factors for all independent variables in the final model were below 2.0

### Comparison of SCL-90-R in occupational groups before and after intervention

**Fig. 1:**
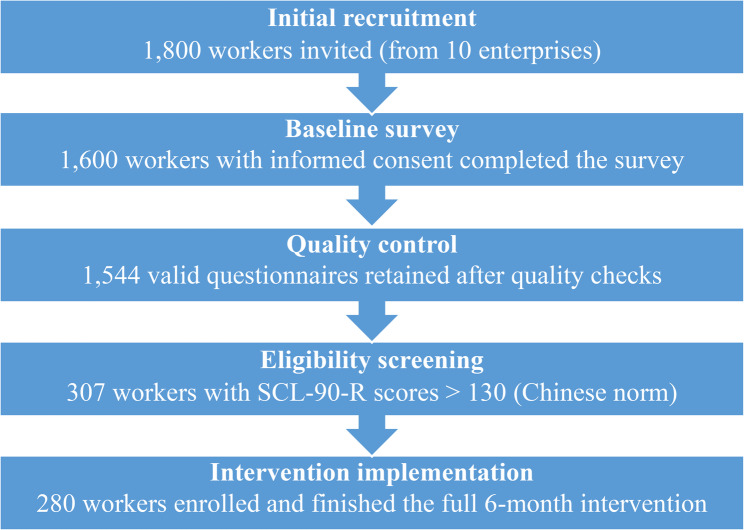
Participant flow diagram from initial recruitment to final intervention completion

**Fig. 2:**
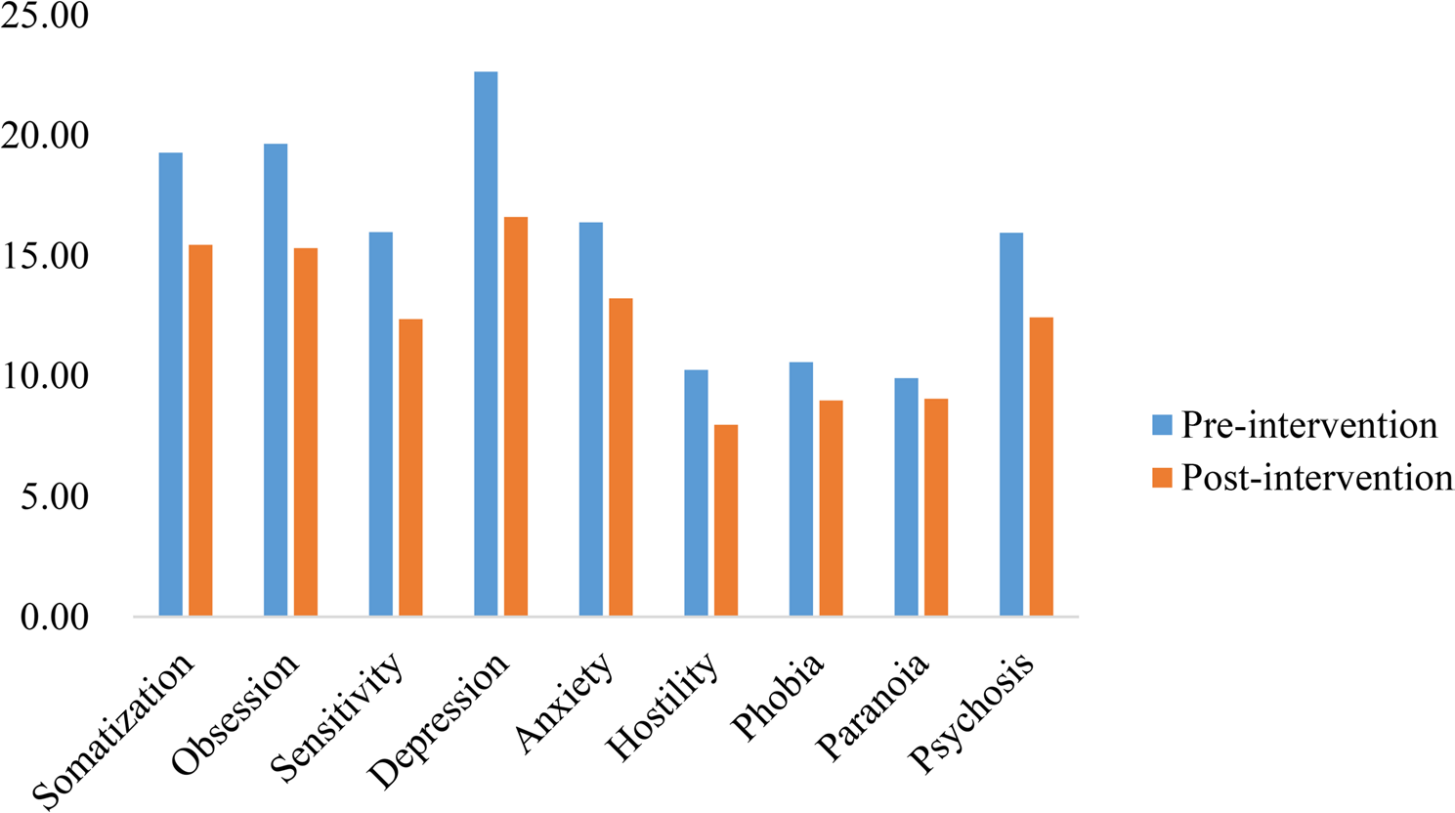
Comparison of SCL-90-R factors before and after intervention

In this survey, 308 individuals had SCL-90-R scores that exceeded the Chinese norm. After the exclusion of invalid questionnaires, 280 individuals, including 198 men and 82 women, with an average age of 32.62 ± 8.73 years and an average length of service of 4.45 ± 3.62 years, voluntarily participated in the full intervention. Participant flow diagram see Fig. 1. Among them, 171 were married, and 109 were unmarried. Intervention research was conducted through group communication, mutual assistance, training, offline communication, and enterprise assistance. The results showed that the total score of the SCL-90-R mental health after intervention was significantly lower than before, from 153.33 ± 20.91 dropped to 121.9 ± 11.43(*P* < 0.001), and the total scores of each factor after intervention were associated with a significant reduction than before(*P* < 0.001). The observed reduction exceeds the minimal clinically important difference, indicating that the improvement is likely meaningful for participants. Details are shown in Fig. 2.

## Discussion

The SCL-90-R is a widely used instrument for evaluating mental disorders and conditions, offering a comprehensive assessment across multiple dimensions—including emotions, thoughts, behaviors, lifestyle habits, interpersonal relationships, diet, and sleep [18, 19]. Introduced in 1973, the original version of this scale was soon revised and validated before being officially published as SCL-90-R in 1976 [16].

The mental health status of the 1,544 migrant workers in the Kunshan’s electronics industry was significantly lower than the Chinese norm (i.e., the general population), indicating lower psychological well-being compared with several other occupational groups surveyed domestically. Specifically, the total SCL-90-R score and the subscale scores for obsessive-compulsive disorder, interpersonal sensitivity, depression, and anxiety were lower in Kunshan than in Shenzhen and Shanghai, but higher than those reported in Shandong [20–22]. The result shows that urbanization levels somewhat influence the mental health of electronics industry employees, with greater psychological pressure observed among electronics factory employees in first-tier cities than those in other cities. Migration itself also appears to exert a substantial impact on mental health; for example, young migrant workers in Shenzhen had poorer mental health than their locally born peers [23]. Globally, depression and anxiety prevalence rates have been reported as high as 38.99% (95% CI = 0.27, 0.51) and 27.31% (95% CI = 0.06,0.58), respectively [24]. Additionally, immigrants from South Korea, Malaysia, and Vietnam exhibit varying degrees of mental health problems [25–27].

Workers in electronics enterprises are exposed to fierce competition in the market economy environment, especially after the end of the COVID-19 pandemic. Drastic market shifts driven by both international and domestic factors have imposed greater demands on workers in terms of knowledge, skills, adaptability, and work efficiency. The resulting “occupational stress” alongside other negative psychosocial factors, poses a threat to workers’ health [28]. Concurrently, traditional occupational hazard factors such as noise, dust, poisons, etc., contribute to physical discomfort among workers and reduce the quality of life and feelings of depression [29]. The present study further demonstrates that exposure to three or more occupational hazard factors directly elevates the risk of mental health problems.

A national health literacy survey conducted in 2022 across 23 industries, involving 340,506 participants, revealed that occupational health literacy levels among the general population tend to increase with age [5]. In contrast, the mental health level of employees in electronics factories exhibited a decline with age (≥45 years) and working years (≥5 years). Contributing factors may include the generally younger age of electronics factory employees, the advantage of piecework wages among young workers, and the high turnover rate in personnel.

A considerable number of worker exhibit diminished mental health as a result of factors such as poor working conditions, heavy workloads, long working hours, and repetitive assembly-line tasks. Consistent with our findings, Do HN et al. demonstrated a significant association between job demands, job control, and depression among electronics factory workers [30]. Furthermore, female workers in this sector tend to report higher levels of anxiety than their male counterparts. Overall, a significant negative correlation between occupational stress and the mental health of workers in electronics manufacturing [31].

The mental health of electronics workers is a critical social issue requiring greater attention. More robust workplace management and mental health education that involve a comprehensive consideration of individual, family and social factors can effectively improve the mental health of workers in electronics factories [32]. This study found that active intervention was associated with improvements among individuals with elevated psychological symptoms, as indicated by reduced SCL-90-R scores following the program. These findings also suggest that it is necessary to establish a sound psychological counselling service system that provides timely mental health support and treatment. Additionally, companies can also reduce employee workload and reduce psychological pressure by promoting employee mental health awareness and peer support to improve the working environment and reduce work intensity. These findings support the need for accessible psychological counselling services and workplace strategies that promote mental health awareness, peer support, and reduced work intensity. Although this study did not evaluate long-term sustainability, scalability, or cost-effectiveness, key components such as peer support and communication training are inherently low-cost and scalable [33, 34]. Future research should prioritize hybrid effectiveness-implementation trials that measure both mental health outcomes and implementation metrics, including cost, reach, and barriers to adoption across different workplaces.

This study uses SCL-90-R to investigate the mental health of urban migrant workers, though it has some shortcomings. First, the results of cross-sectional surveys are time-bound. Secondly, the on-site questionnaire survey may be subject to response bias. The SCL-90 questionnaire contains a large number of questions, and interviewees may provide inaccurate information or misunderstand questions. Mean imputation for missing items may introduce bias by reducing variance and potentially attenuating associations. Thirdly, the adaptation of the intervention study may be inadequate and may not provide a comprehensive view because it relies primarily on voluntary participants. Finally, the lack of a control group limits causal inference, as we cannot rule out Hawthorne effects or natural variation. Additionally, this intervention study was not prospectively registered in a clinical trial registry, as it was conducted within routine occupational health surveillance rather than as a formal trial, and no formal a priori sample size calculation was conducted, however, the observed effect size (Cohen’s *d* ≈ 1.5) substantially exceeds conventional thresholds, suggesting that the findings are robust despite this limitation. We acknowledge that prospective registration enhances transparency and recommend it for future confirmatory studies. This design was chosen for practical and ethical reasons. Future studies should adopt randomized or stepped-wedge designs to better establish efficacy, develop digital investigation methods, streamline survey content, and create culturally specific peer support programs to overcome sample size limitations and promote effective mental health practices.

## Conclusion

In summary, research on the mental health factors of workers in electronics factories offers important practical implication for analysis of the mental health status of manufacturing workers and for the protection of their mental health. Mental health issues are known as common social problems. In counseling migrant workers in electronic enterprises, attention should be given to both occupational health and psychological problems. Comprehensive psychological intervention measures should be promoted to eliminate negative emotions and psychological barriers among this group.

## Supplementary Information

Supplementary Material 1.

## Data Availability

No datasets were generated or analysed during the current study.

## Abbreviations

SCL: 90-R Symptom Check list 90 Revised
COVID: 19 Corona Virus Disease 2019
DMF N,N: Dimethylformamide

## References

[1] Gondek D, Bernardi L. Mental Health and Wellbeing of Population with Migrant Background in Switzerland - a Scoping Review and Evidence Map of Quantitative Evidence. J Immigr Minor Health. 2023;25(5):1108–17. 37237054 10.1007/s10903-023-01490-5 PMC10509096

[2] Salas-Wright CP, Maldonado-Molina MM, Pérez-Gómez A, et al. The Venezuelan diaspora: Migration-related experiences and mental health. Curr Opin Psychol. 2022;47:101430. 35985072 10.1016/j.copsyc.2022.101430 PMC9870179

[3] Lebano A, Hamed S, Bradby H, et al. Migrants’ and refugees’ health status and healthcare in Europe: a scoping literature review. BMC Public Health. 2020;20(1):1039. 32605605 10.1186/s12889-020-08749-8 PMC7329528

[4] National Health Commission of. the People’s Republic of China, Statistical Bulletin on the Development of Health Undertakings in 2023.

[5] Sun YY, Sun X, Wan X, et al. Occupational health literacy level and its influencing factors among key populations in China in 2022. Chin Occup Med. 2023;50(03):241–7.

[6] Liu Y, Shen HX, Song XP, et al. Survey on occupational health status of 5328 enterprises in Kunshan. Chin J Industrial Med. 2019;36(04):346–8.

[7] Koh YS, et al. Depressive and anxiety symptoms among migrant workers and migrant domestic workers in Singapore. Ann Acad Med Singap. 2025;55(2):74–85. 41773818 10.47102/annals-acadmedsg.2025251

[8] Sznajder KK, Harlow SD, Wang J, et al. Factors associated with symptoms of poor mental health among women factory workers in China’s supply chain. Int Arch Occup Environ Health. 2022;95(6):1209–19. 35001196 10.1007/s00420-021-01820-w PMC8743097

[9] Wang WK, Wang MT, Pan H, et al. Health risky behaviors among rural-to-urban migrant workers in China: prevalence, patterns, and association with distal and proximal factors. Front Public Health. 2025;13:1459661. 40061463 10.3389/fpubh.2025.1459661 PMC11885242

[10] Caxaj CS, Shkopi E, Naranjo CT, et al. Health, social and legal supports for migrant agricultural workers in France, Italy, Spain, Germany, Canada, Australia and New Zealand: a scoping review. Front Public Health. 2023;11:1182816. 37869183 10.3389/fpubh.2023.1182816 PMC10588640

[11] Urrego-Parra HN, Rodriguez-Guerrero LA, Pastells-Peiro R et al. The Health of Migrant Agricultural Workers in Europe: A Scoping Review. J Immigr Minor Health. 2022;24(6):1580–9. 10.1007/s10903-022-01330-y 35133580

[12] Hynie M, Oda A, Calaresu M, et al. Access to Virtual Mental Healthcare and Support for Refugee and Immigrant Groups: A Scoping Review. J Immigr Minor Health. 2023;25(5):1171–95. 37407884 10.1007/s10903-023-01521-1 PMC10509103

[13] Ho KHM, Yang C, Leung AKY. Peer Support and Mental Health of Migrant Domestic Workers: A Scoping Review. Int J Environ Res Public Health. 2022;19(13):7617. 35805278 10.3390/ijerph19137617 PMC9265321

[14] Schmitz N, Hartkamp N, Kiuse J, et al. The Symptom Check-List-90-R (SCL-90-R): a German validation study. Qual Life Res. 2000;9(2):185–93. 10983482 10.1023/a:1008931926181

[15] Carrozzino D, Christensen KS, Patierno C, et al. The Hopkins Symptom Checklist (SCL-90-R): A Patient-Reported Outcome Measure in Parkinson’s Disease. J Geriatr Psychiatry Neurol. 2022;35(5):689–97. 34971324 10.1177/08919887211060020

[16] Derogatis LR, Rickels K, Rock AF. The SCL-90 and the MMPI: a step in the validation of a new self-report scale. Br J Psychiatry. 1976;128:280–9. 1252693 10.1192/bjp.128.3.280

[17] Tong HJ. A study on the 20-year change of SCL-90 and its norms. Psychol Sci. 2010;33(04):928–30.

[18] Yilmaz A, Gokcen P, Yilmaz H, et al. Irritable Bowel Syndrome in Dialysis Patients and Symptom Check List Revised (SCL 90-R) Screening. Eurasian J Med. 2021;53(3):220–6. 35110100 10.5152/eurasianjmed.2021.20412 PMC9879226

[19] Yu Y, Wan C, Zhao X, et al. Undergraduate students’ norms for the Chinese version of the symptom check-List-90-R (SCL-90-R). BMC Public Health. 2020;20(1):1588. 33087089 10.1186/s12889-020-09689-z PMC7579932

[20] Zhang LH, Li AG, Liu CX. Investigation and analysis of mental health status of migrant workers in Shenzhen. Hainan Med. 2017;28(01):151–2.

[21] Li ZS, Wang SY, Lu YL et al. The characteristics and relationship between coping styles and mental health of migrant workers. Mod Prev Med. 2013;40(04):690–3.

[22] Qi XQ, Zou JF, Fan ZB et al. Survey on occupational and mental health of migrant women workers in pharmaceutical and electronics industries in Shandong Province. Occup Health. 2012;28(07):769–72.

[23] Zhong BL, Liu TB, Chan S et al. Common mental health problems in rural-to-urban migrant workers in Shenzhen, China: prevalence and risk factors. Epidemiol Psychiatr Sci. 2018;27(3):256–65. 10.1017/S2045796016001141 PMC6998856 28067189

[24] Hasan SI, Yee A, Rinaldi A et al. Prevalence of common mental health issues among migrant workers: A systematic review and meta-analysis. PLoS One. 2021;16(12):e260221. 10.1371/journal.pone.0260221 PMC8638981 34855800

[25] Kim S, Lee DJ, Kim SH et al. The Health Status and Management of Migrant Workers in Cheonan: A Comparison Study With Korean Citizens. J Korean Med Sci. 2023;38(46):e398. 10.3346/jkms.2023.38.e398 PMC10681839 38013650

[26] Htay M, Latt SS, Maung KS et al. Mental Well-Being and Its Associated Factors Among Myanmar Migrant Workers in Penang, Malaysia. Asia Pac J Public Health. 2020;32(6–7):320–7. 10.1177/1010539520940199 32672053

[27] Giri B, et al. Mental Health of Nepalese Migrant Workers: A Call for Action in South Korea. JNMA J Nepal Med Assoc. 2025;63(288):636–40. 41783665 10.31729/jnma.9165 PMC12827870

[28] Yan T, Ji F, Bi M, et al. Occupational stress and associated risk factors among 13,867 industrial workers in China. Front Public Health. 2022;10:945902. 36466474 10.3389/fpubh.2022.945902 PMC9714303

[29] Febriyanto K, Rahman FF, Guedes J. The physical and psychological effects of occupational noise among seafarers: a systematic review. Int J Environ Health Res. 2023;1–13. 10.1080/09603123.2023.2266703 37820712

[30] Do HN, Nguyen AT, Nguyen H, et al. Depressive Symptoms, Suicidal Ideation, and Mental Health Service Use of Industrial Workers: Evidence from Vietnam. Int J Environ Res Public Health. 2020;17(8):2929. 32340335 10.3390/ijerph17082929 PMC7216084

[31] Lu Y, Liu Q, Yan H et al. Effects of occupational hazards and occupational stress on job burn-out of factory workers and miners in Urumqi: a propensity score-matched cross-sectional study. BMJ Open. 2022;12(9):e51911. 10.1136/bmjopen-2021-051911 PMC9462083 36647785

[32] Mo P, Cheng Y, Lau J. Work-related factors on mental health among migrant factory workers in china: Application of the Demand-Control and Effort-Reward Imbalance Model. Health Soc Care Community. 2022;30(2):656–67. 10.1111/hsc.13176 32989898

[33] Thayyilayil SA et al. Culturally Adapted Mental Health Education Programs for Migrant Populations: A Scoping Review. Int J Environ Res Public Health. 2026;23(1). 10.3390/ijerph23010072 PMC12841287 41595866

[34] Cameron G, et al. Effectiveness of Digital Mental Health Interventions in the Workplace: Umbrella Review of Systematic Reviews. JMIR Ment Health. 2025;12:e67785. 39854722 10.2196/67785 PMC11806266

