# Supplementary material for: Investigation and intervention of psychological factors among migrant workers in electronics enterprises in Kunshan, China
Source: BMC Public Health. 2026 Apr 14;26:1666. doi: 10.1186/s12889-026-27359-4 (PMC13196165; doi:10.1186/s12889-026-27359-4)
Supplement: Supplementary file 1 — Supplementary Material 1. [file 12889_2026_27359_MOESM1_ESM.docx]

General information questionnaire (mark "√" in the options)

Explanation: This questionnaire aims to understand your general situation, and we will keep the information you fill out strictly confidential! Please rest assured to answer!

Number date

Name, gender, age, ethnicity

1. What is your place of origin? Within province A and outside province B

2. What is your cultural level? A Junior High School B High School (or Vocational College) C College D Undergraduate E Undergraduate or above

3. Your marital status? A unmarried B married C divorced

4. What profession are you currently engaged in? A physical labor B mental labor

5. How much time do you work per day? A 8 hours a day B 12 hours a day C 12 hours or more

How often do you ask to go home? A once a year B twice a year C three times a year D three times a year or more

7. Who are you currently living with? A parents B spouses C collective dormitories D renting their own rooms

8. What is your current monthly income? A<1500 yuan B1500-2000 yuan C2000 yuan D2500-3000 yuan E3000 yuan

Above yuan

Can your salary be paid on time? A Yes B No

10. You spend approximately () yuan per month on your diet.

The average annual cost you spend on clothing is approximately () yuan.

The annual cost you spend on transportation is approximately () yuan.

The monthly cost you spend on communication is approximately () yuan.

14. Your monthly deposit is approximately () yuan.

The monthly cost you spend on housing is () yuan.

16. Your spare time is mainly used for A watching TV and surfing the internet, B going to school and studying in your spare time, C sleeping, and D other activities

17. Does your company have social insurance coverage for you? A Yes B No

Do you often participate in group activities? A never participates, B occasionally participates, C frequently participates, and D actively participates

19. Your social circle is A, fellow villager B, integrated into society C, without social interaction

Do you feel that your place of residence is safe? A Can B No C General

21. Do you care about national policies and social news? A Yes B No

22. How is your relationship with your direct supervisor? A is good, B is average, and C is not good.

23. Your purpose of coming here to work is A to earn money (to support your family) B to increase your experience C to improve yourself

Do you think you are from Kunshan? A is B, no

How long have you been working here? A within one year B1-2 years C2 years D3 years or more

26. Your way of working is A working during idle farming hours B working outside all year round

27. Do you have your own house in Kunshan? A has B has no

Do you feel that there is room for improvement in your current unit? A has B has no

29. What are your plans in the future? A continues to work, B starts his own business, C goes back to his hometown, and D goes to another city
